# Supplementary material for: Molecular Insights into the Marine Gastropod Olivancillaria urceus: Transcriptomic and Proteopeptidomic Approaches Reveal Polypeptides with Putative Therapeutic Potential
Source: Int J Mol Sci. 2025 Apr 16;26(8):3751. doi: 10.3390/ijms26083751 (PMC12027567; doi:10.3390/ijms26083751)
Supplement: Supplementary file 1 [file ijms-26-03751-s001.zip › Supplementary Figure S1.pdf]

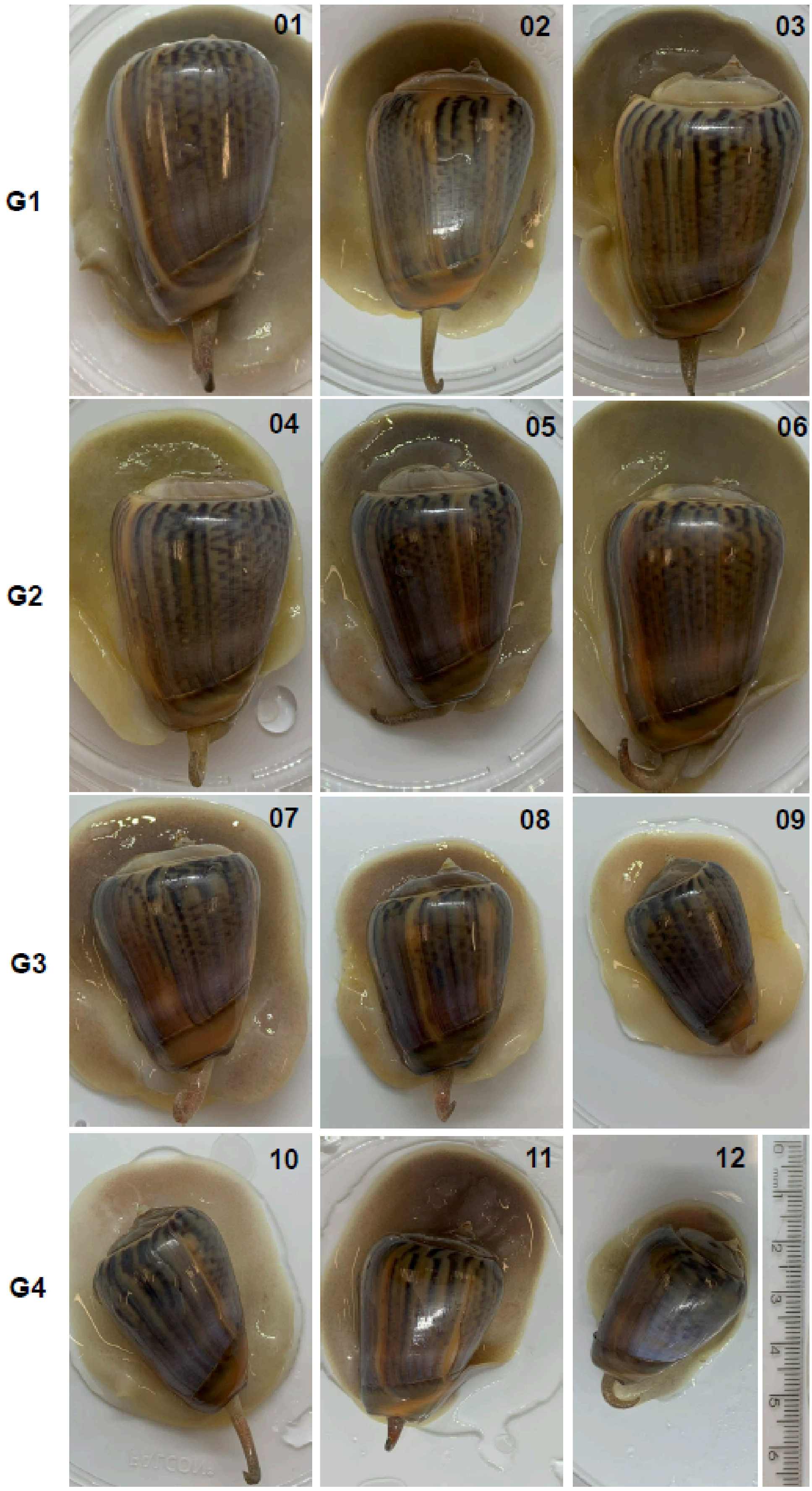

Supplementary Figure S1. For proteomics and peptidomics, a total of 12 specimens were used, and they were divided into four groups (G1, G2, G3 and G4) based on shell size.
