# Supplementary material for: Molecular Insights into the Marine Gastropod Olivancillaria urceus: Transcriptomic and Proteopeptidomic Approaches Reveal Polypeptides with Putative Therapeutic Potential
Source: Int J Mol Sci. 2025 Apr 16;26(8):3751. doi: 10.3390/ijms26083751 (PMC12027567; doi:10.3390/ijms26083751)
Supplement: Supplementary file 1 [file ijms-26-03751-s001.zip › Supplementary Figure S3.pdf]

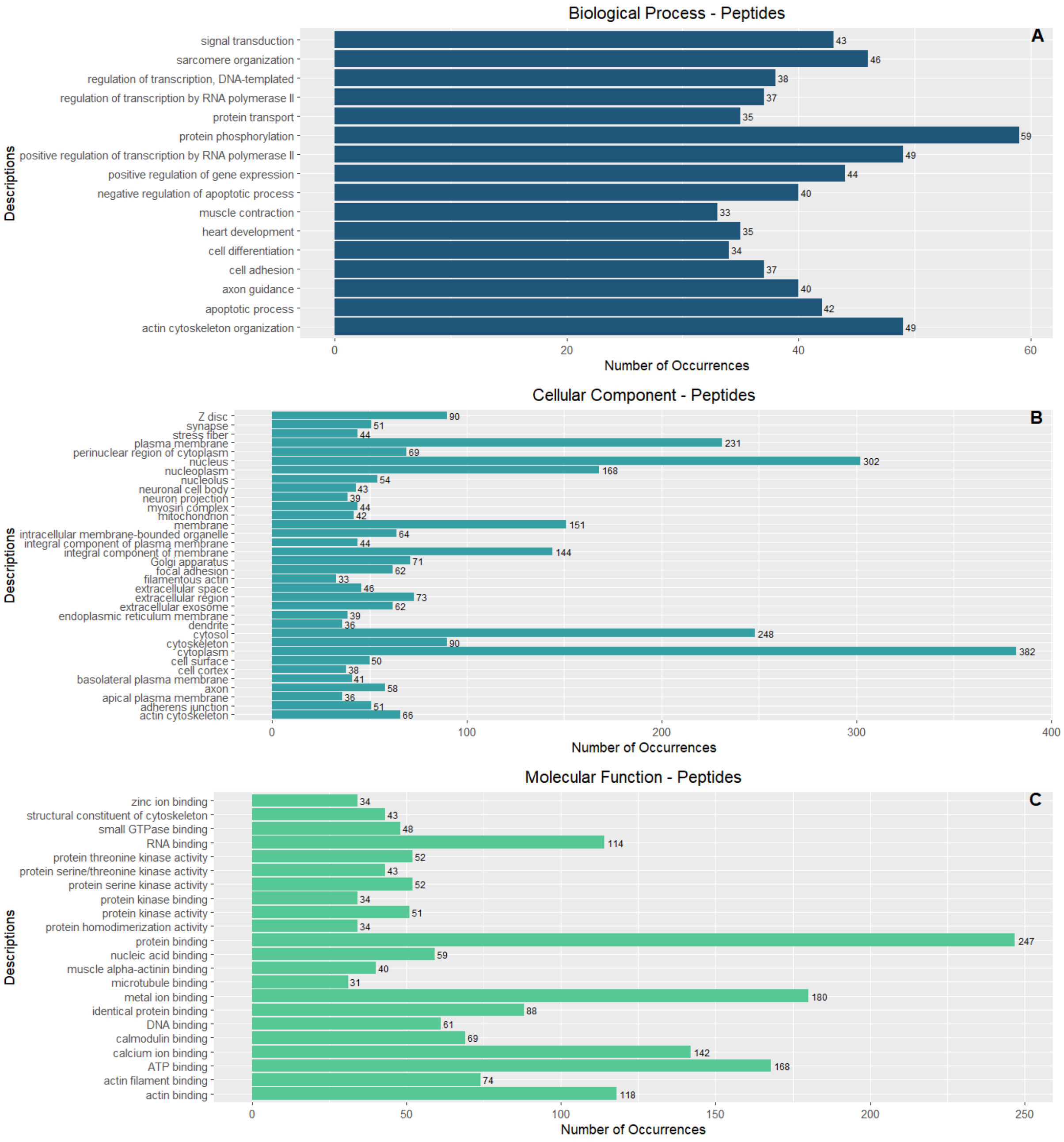

Supplementary Figure S3. Distribution of Gene Ontology (GO) terms for the peptidome of *Olivancillaria urceus*, classified into the categories of Biological Process (A), Cellular Component (B), and Molecular Function (C).The bar charts indicate the number of occurrences for each GO term associated with the identified peptides.
