# Supplementary material for: Molecular Insights into the Marine Gastropod Olivancillaria urceus: Transcriptomic and Proteopeptidomic Approaches Reveal Polypeptides with Putative Therapeutic Potential
Source: Int J Mol Sci. 2025 Apr 16;26(8):3751. doi: 10.3390/ijms26083751 (PMC12027567; doi:10.3390/ijms26083751)
Supplement: Supplementary file 1 [file ijms-26-03751-s001.zip › Supplementary Material S1_Statistical Results.pdf]

Supplementary Material 1

Results Statistics of Proteomic experiments

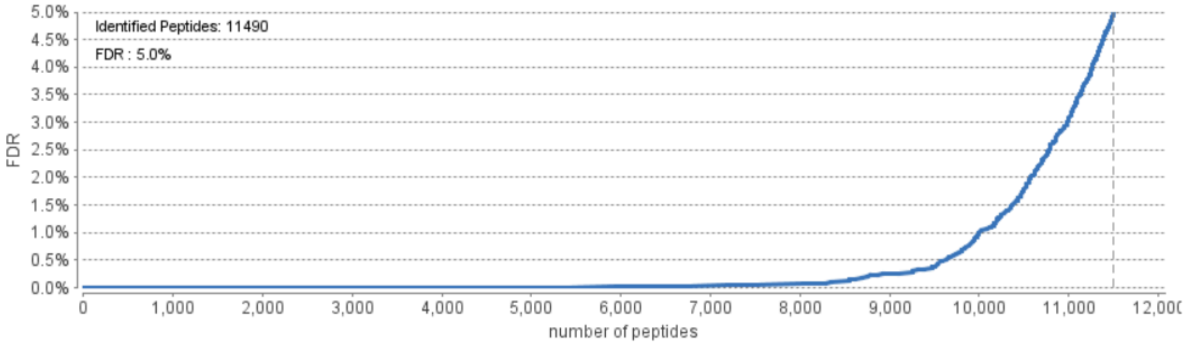

Figure 1. False discovery rate (FDR) curve. X axis is the number of peptides being kept. Y axis is the corresponding FDR. 11490 peptides: FDR 1.1- 5%= 1488 peptides (13%); FDR 0.11-1%=1659 peptides (14.4%) FDR ≤ 0.1% (72.6%)

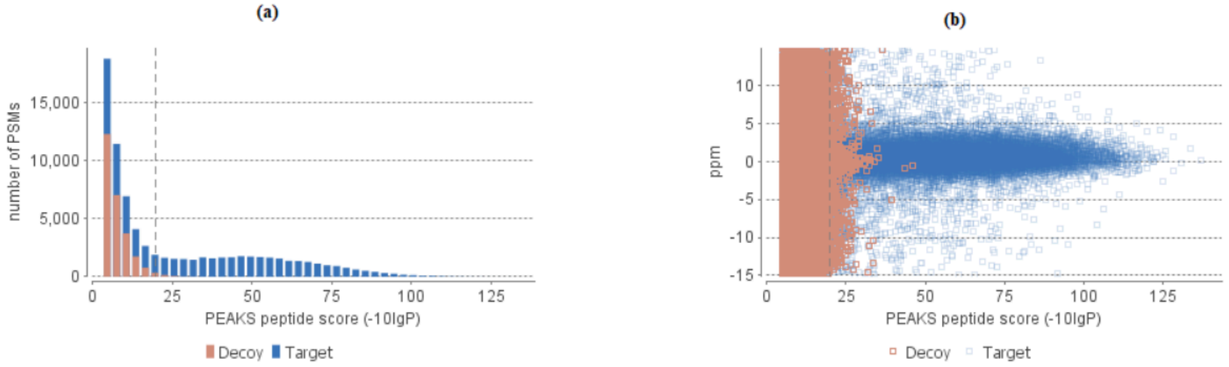

Figure 2. PSM score distribution. (a) Distribution of PEAKS peptide score; (b)Scatterplot of Peaks peptide score versus precursor mass error.

Table 1. Statistic of data

	#Scans		#Features	Identified			#Peptides	#Sequences	#Proteins*		
	MS1	MS/MS		#PSMs	#Scans	#Features**			Groups	All	Top
Total	13063	175922	181934	33128	33128	26675	11490	10812	1337	3185	2179
proteômica 1	3730	43038	43183	7737	7737	6046	3944	3738	809	1966	1338
proteômica 2	3191	44463	46317	8797	8797	7176	4698	4434	896	2147	1458
proteômica 3	3071	43968	50912	8516	8516	7047	4806	4529	818	2002	1370
proteômica 4	3071	44453	41522	8078	8078	6406	4460	4232	703	1715	1191

* proteins with significant peptides are used in counts.

** features are identified by DB search only.

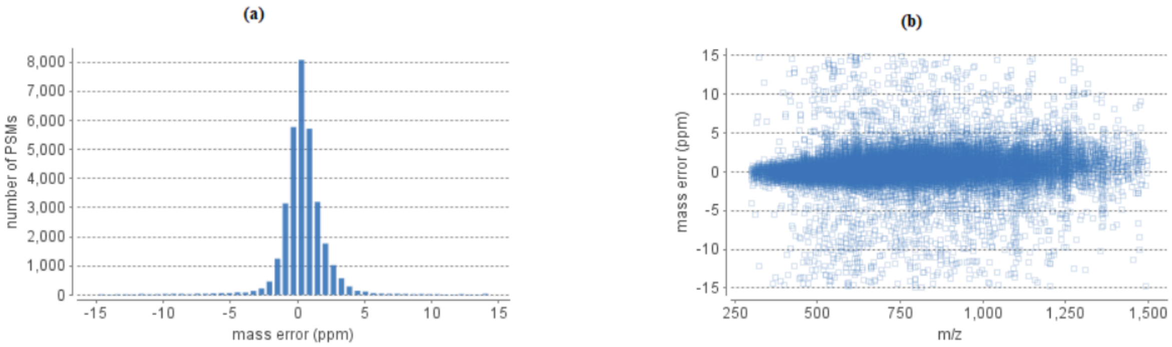

Figure 3. Precursor mass error of peptide-spectrum matches (PSM) in filtered result. **(a)** Distribution of precursor mass error in ppm; **(b)** Scatterplot of precursor m/z versus precursor mass error in ppm.

<u>Parameters</u>

FDR <5%

Peptide -10lgP ≥19.8

Protein -10lgP ≥20

Search Engine Name: PEAKS

Parent Mass Error Tolerance: 15.0 ppm

Fragment Mass Error Tolerance: 0.5 Da

Precursor Mass Search Type: monoisotopic

Enzyme: Trypsin

Max Missed Cleavages: 3

Results Statistics of Peptidomic experiments

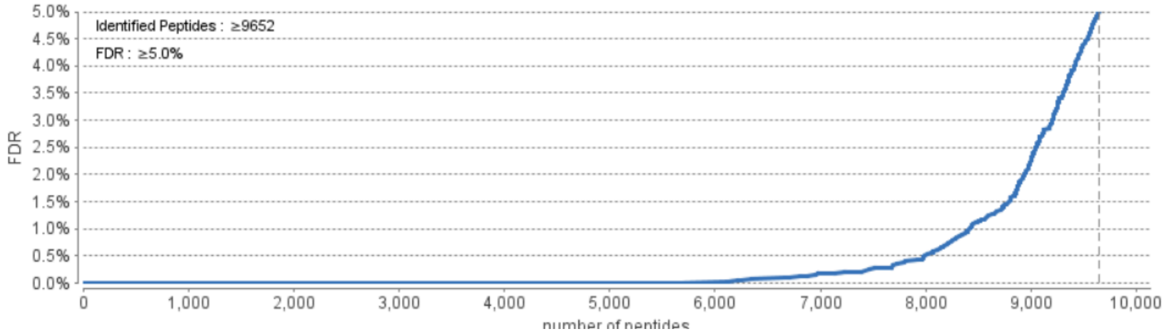

Figure 1. False discovery rate (FDR) curve. X axis is the number of peptides being kept. Y axis is the

corresponding FDR. 9652 peptides: FDR 1.1- 5%= 1223 peptides (12.7%); FDR 0.11-1%=1697 peptides (17.6%) FDR <0.1%= 6732 peptides (72.6%).

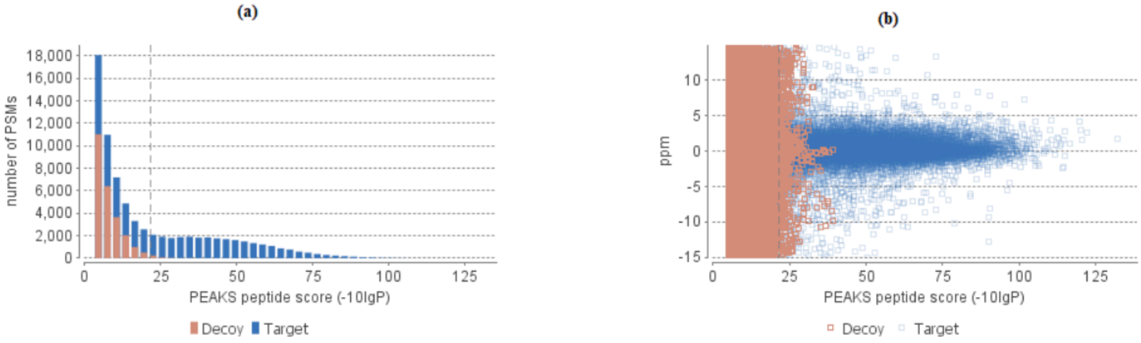

Figure 2. PSM score distribution. (a) Distribution of PEAKS peptide score; (b)Scatterplot of Peaks peptide score versus precursor mass error.

Table 1. Statistic of data

	#Scans		#Features	Identified			#Peptides	#Sequences	#Proteins*		
	MS1	MS/MS		#PSMs	#Scans	#Features**			Groups	All	Top
Total	12225	176091	138665	28129	28129	23734	9663	8886	906	2144	1484
peptidômica 1	3052	44419	32738	7044	7044	5697	4464	4216	463	1156	803
peptidômica 2	3093	43873	38377	8625	8625	7360	5690	5357	529	1323	921
peptidômica 3	3045	43869	34737	6554	6554	5631	4486	4156	480	1223	852
peptidômica 4	3035	43930	32813	5906	5906	5046	4064	3765	461	1183	829

* proteins with significant peptides are used in counts.

** features are identified by DB search only.

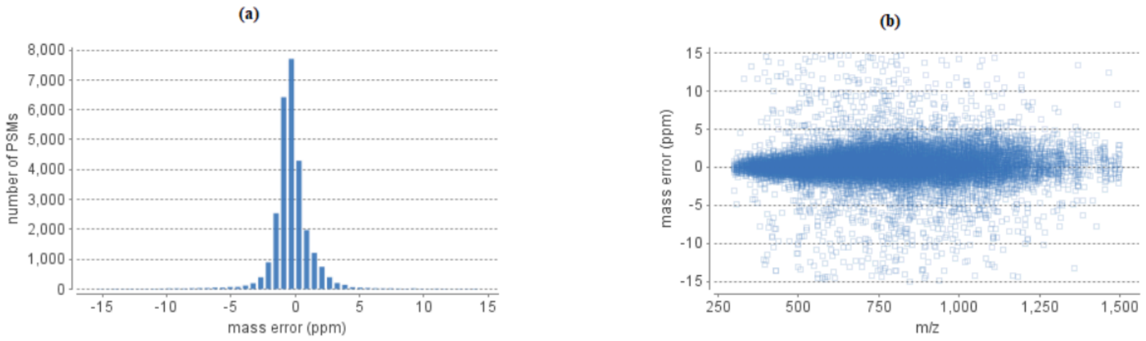

Figure 3. Precursor mass error of peptide-spectrum matches (PSM) in filtered result. **(a)** Distribution of precursor mass error in ppm; **(b)** Scatterplot of precursor m/z versus precursor mass error in ppm.

<u>Parameters</u>

FDR <5%

Peptide -10lgP ≥19.8

Protein -10lgP ≥20

Search Engine Name: PEAKS

Parent Mass Error Tolerance: 15.0 ppm

Fragment Mass Error Tolerance: 0.5 Da

Precursor Mass Search Type: monoisotopic

Enzyme: none
